# Industrial processing of Ecuadorian household food acquisitions according to NOVA, socioeconomic status and territory

**DOI:** 10.3389/fpubh.2026.1970719

**Published:** 2026-10-12

**Authors:** Orlando Meneses Quelal

**Affiliations:** Carrera de Alimentos, Universidad Politécnica Estatal del Carchi (UPEC), Tulcán, Carchi, Ecuador

**Keywords:** ENIGHUR, food systems, household expenditure, household food acquisitions, multivariate analysis, ultra-processed foods

## Abstract

**Introduction:**

Household food-acquisition data can characterise the technological structure of food systems without equating monetary expenditure with individual dietary intake. This study quantified the monetary distribution of Ecuadorian household food acquisitions according to NOVA and examined socioeconomic and territorial patterning.

**Methods:**

We analysed 35,398 households from the 2024–2025 Ecuadorian National Survey of Household Income and Expenditure (ENIGHUR), accounting for survey weights, strata and primary sampling units. Of 899 detailed food and non-alcoholic beverage fields, 840 recorded positive expenditure. Products were classified using NOVA through an explicit uncertainty audit, with fourteen categories retained as REVIEW. National and domain estimates, survey-weighted fractional logit models, sensitivity analyses and weighted principal component analysis were performed.

**Results:**

Weighted national expenditure shares were 72.91% for NOVA 1, 3.11% for NOVA 2, 11.74% for NOVA 3 and 12.25% for NOVA 4. The NOVA 4 share increased from 9.82% in the first income decile to 15.94% in the tenth. Fractional logit modelling confirmed a positive association with income and a significant income-by-urban-residence interaction, with greater urban divergence at higher income levels. Alternative classification of REVIEW items changed the national NOVA 4 estimate only from 12.21% to 12.53%. Weighted PCA retained three components explaining 54.44% of variance.

**Discussion:**

Minimally processed foods remained the dominant component of household monetary acquisitions, while NOVA 4 products were widely present and showed clear socioeconomic and territorial patterning. The small sensitivity range supports the robustness of the classification. These findings provide a reproducible national baseline for monitoring changes in Ecuadorian household food acquisitions by degree of industrial processing.

## 1 Introduction

The transformation of food systems is expressed not only through changes in the amount of food available, but also through modifications in the technological organisation of the products that reach homes. Industrial processing can extend shelf life, improve microbiological safety, reduce preparation times, and enable distribution through extensive supply chains (1). Therefore, processing in itself does not constitute an adverse property. The analytical question lies in identifying for what purpose a food is processed, what technological modifications are incorporated and to what extent these modifications reorganize the household acquisition structure. The NOVA system was developed to operationalize this perspective through a classification based on the nature, extent and purpose of processing, differentiating it from classifications focused exclusively on nutrients (2). Martinez-Steele et al. (3) later specified that a reproducible application requires item-level adjudication, explicit rules, and transparent treatment of ambiguous products. These requirements are particularly relevant when the source of information is an expenditure survey, because the official product description may be detailed, but does not necessarily incorporate the complete list of ingredients.

The expansion of industrially formulated products has turned the degree of processing into a relevant dimension of the contemporary food transition. Baker et al. (4) showed that the growth of ultra-processed foods is related to simultaneous modifications in manufacturing, marketing, advertising, distribution, and organisation of domestic time. Popkin and Ng (5) pointed out that a transition toward diets dominated by ultra-processed products does not represent an inevitable consequence of economic growth, but rather an outcome conditioned by market structures and food policy decisions. Internationally, sales of ultra-processed foods and beverages have increased unevenly depending on the level of national income and the maturity of the retail system (6). This heterogeneity implies that the percentage of ultra-processed products observed in a country must be interpreted within the specific trajectory of its food system and not as a universal threshold of modernisation. Recent literature has reinforced this systems-level interpretation by situating ultra-processing in the interaction between industrial formulation, commercial environments, purchasing behaviour and regulation (7, 8).

Household acquisition and expenditure surveys are a particularly suitable source for studying this dimension of the food system because they record how monetary resources are allocated between product groups. The construct measured differs from an individual 24-h dietary recall. Vepsäläinen et al. (9) showed that food purchase data correlate with intake for several groups, but both measures are not interchangeable, given that purchased products can be stored, wasted, shared, or consumed by different members of the household. This difference constitutes an advantage when the scientific question refers to the purchasing basket exposed to the market. Louzada et al. (10) demonstrated that household acquisition surveys can be used to characterise the availability of ultra-processed foods as long as the estimand is defined consistently with the source. In Brazil, series of household surveys have identified long-term changes in the processing profile of food availability (11), while Australian purchasing panels have revealed socioeconomic differences in the acquisition of ultra-processed products (12).

Socioeconomic patterning deserves particular attention because the direction of the relationship between socioeconomic position and exposure to ultra-processed products is not universal. Dicken et al. (13) reviewed nationally representative studies and showed that gradients change between countries and stages of food transition. In many high-income contexts, socially disadvantaged groups have greater exposure to ultra-processed products. In certain middle-income countries, however, greater purchasing power can still facilitate access to packaged goods, industrial beverages, food services, and convenience foods. Coyle et al. (12) identified a socioeconomic gradient in a mature Australian retail market, while studies conducted in transforming systems show different configurations. Considering socioeconomic position only as a control variable would hide a central part of the phenomenon. In food systems in transition, income can modify both the level of acquisition of ultra-processed products and the way in which urbanisation is expressed in the household basket.

Ecuador has a specific regulatory trajectory within this regional context. The country implemented a traffic light front labelling system before several Latin American countries adopted warning seals. Freire et al. (14) documented that Ecuadorian consumers recognize and use the nutritional traffic light, although their interpretation and behavioural response vary between groups. Sarasty et al. (15) subsequently analysed the effects of the system on processed foods and the market response, while Cordero-Ahiman et al. (16) examined dimensions linked to the use and interpretation of nutritional information. At the same time, Ecuadorian studies have shown dietary changes in particular territories, such as the Galapagos, where transformations in food availability coexist with a high prevalence of excess weight (17). More recently, Freire et al. (18) adapted and validated the NOVA 27 screening for Ecuador, demonstrating that the measurement based on the degree of processing is operationally viable in the national context.

Against this background, the specific gap addressed by the present study is the national characterisation of the monetary structure of household food acquisitions according to NOVA using the 2024–2025 ENIGHUR, together with its socioeconomic and territorial patterning, while preserving classification uncertainty and complex-survey inference. This question is distinct from individual dietary screening: the ENIGHUR estimand is the monetary composition of household acquisitions, not food intake. The survey provides detailed standardised expenditure fields together with income, demographic and territorial variables under a probabilistic national design, allowing these dimensions to be examined within a single population framework (14, 17, 18, 19).

The scientific application of NOVA requires, however, methodological precision. Sadler et al. (20) demonstrated that processing classification systems differ in objectives, criteria, and operational boundaries. Braesco et al. (21) highlighted reproducibility problems when commercial or administrative descriptions do not make it possible to know the formulation with certainty. Sneed et al. (22) verified that the assignment of processing categories to dietary recall data can reach adequate levels of reliability if a structured protocol is used, although uncertainty increases in compound or incompletely described foods. Price et al. (23) further showed that alternative decisions about certain whole grain products can modify estimated cardiometabolic associations. From these observations emerges a central methodological principle for the present study: a national household-acquisition database should not convert ambiguous categories into apparently exact classifications through automatic, undocumented rules. Uncertainty must be preserved, quantified and subjected to sensitivity analysis.

The novelty of the present study is based on a hierarchical analytical architecture. First, standardised official monetary spending is used and not an *ad hoc* reconstruction. Second, technological classification is carried out field by field and explicitly preserves the uncertainty of products whose description does not allow a single adjudication. Third, national estimates, domain analyses, and fractional response modelling incorporate the complex survey design, so that inference remains at the population scale for which ENIGHUR was constructed. Fourth, a weighted multivariate characterisation of eleven spending dimensions is used solely as a structural complement to identify axes of covariation that are not visible in a single NOVA share. This hierarchy keeps the technological structure and its socioeconomic gradients as the main question and uses dimensional reduction to contextualize, not replace, the central estimates.

The objectives were to quantify the monetary shares of Ecuadorian household food acquisitions assigned to the four NOVA groups, with particular emphasis on NOVA 4; characterise their socioeconomic and territorial patterning; estimate adjusted associations using a model consistent with both the fractional response and the complex survey design; identify the products contributing most to NOVA 4 expenditure; assess sensitivity to ambiguously classified categories; and, as a complementary objective, describe the principal axes of covariation across eleven food-expenditure dimensions using weighted principal component analysis. We hypothesised that the NOVA 4 share would increase across the income distribution, that the urban–rural contrast would vary by income level, that regional differences would persist after adjustment, and that classification uncertainty would have only a limited effect on the national estimate. The study was designed to provide a reproducible baseline of the technological structure of food acquisitions within the Ecuadorian food system.

## 2 Materials and methods

### 2.1 Study design and data source

Following the quantitative research-design framework of Creswell and Creswell (24), this study used a quantitative approach, a descriptive-analytical scope, and an observational, non-experimental cross-sectional design based on secondary microdata. We analysed the 2024–2025 Ecuadorian National Survey of Household Income and Expenditure (ENIGHUR), implemented by Ecuador’s National Institute of Statistics and Censuses. The target population comprises households and their members living in private dwellings in Ecuador, and the survey used a probabilistic, stratified, two-stage design in which primary sampling units were selected first and occupied private dwellings second (19).

Sample size was not independently recalculated for the present secondary analysis because all households with valid records in the released file were retained. At the ENIGHUR design stage, INEC used household monetary food expenditure as the design variable and calculated the dwelling sample separately by domain as nₖ = [n₀,ₖ/(1 + n₀,ₖ/Nₖ)] × [1/(1 − TNR)], where n₀,ₖ = Z^2^₍₁₋α/₂₎Sₖ^2^/(εₖ^2^μₖ^2^) and Sₖ^2^ = σₖ^2^DEFFₖ. The calculation incorporated finite-population correction, design effect, a 95% confidence level, domain-specific relative margins of error and an allowance for non-response; 12 dwellings were then selected per primary sampling unit. The resulting planned sample was 41,184 dwellings (3,432 primary sampling units × 12), while the released analytical file used here contained 35,398 households with valid records (19).

### 2.2 Analytical files and household integration

The quantitative construction was based on two official ENIGHUR components. The file ENIGHUR25_HOGARES_AGREGADOS.csv provided survey identifiers, demographic characteristics, current income per capita, national income deciles, natural region, area of residence, and expenditure aggregates. The file ENIGHUR2025_GASTOS_HMO.csv provided the detailed standardised monetary expense fields by product. This second file was used as the primary source of food expenditure because it incorporates the standardisation developed in the official ENIGHUR flow and therefore avoids additional analytical reconstructions from daily values. The food and non-alcoholic beverage block comprised 899 detailed fields. Of them, 840 recorded positive expenditure in at least one home. The sum of these fields exactly reproduced, within numerical precision, the official aggregate d1 of foods and non-alcoholic beverages in each of the 35,398 households.

The unit of analysis was the home. The variable Identif_hog was used as a deterministic key between the aggregate file and the standardised expenditure file. All household archive records were preserved. Sorting processing was performed on the detailed fields before calculating NOVA totals for each household. For each household, the monetary values of the fields assigned to NOVA 1, NOVA 2, NOVA 3 and NOVA 4 were added. Each detailed field received a single analytical status: NOVA 1, NOVA 2, NOVA 3, NOVA 4, REVIEW or EXCLUDED. This structure avoided double counting and made it possible to reconcile the technological regrouping with the official total.

### 2.3 Technological classification of products

The classification followed NOVA conceptual definitions, which distinguish unprocessed or minimally processed foods, processed culinary ingredients, processed foods, and ultra-processed foods (2). The operating principles described by Martinez-Steele et al. (3) were applied, with direct use of the official name of the product, explicit justification and preservation of uncertainty. NOVA 1 included foods described as fresh, refrigerated, frozen, dried, pasteurised, or subjected to minimal transformations without evidence of formulation. NOVA 2 comprised culinary ingredients such as oils, fats, sugars and salt when its primary function was domestic preparation. NOVA 3 included recognisable foods preserved or made primarily by incorporating culinary ingredients. NOVA 4 included industrial formulations and characteristic categories, including soft drinks, confectionery, mass-produced packaged baking products, certain flavoured dairy products, instant preparations and equivalent products when the official description supported the classification. The classification was based on the technological purpose of processing and not on a nutritional density score.

Before running any statistical analysis, a product-level audit file was constructed. Each field was linked to its official description, observed monetary expenditure, proposed NOVA category, analytical scope, confidence level, decision rationale, and sensitivity scenarios where applicable. The classification was contrasted with the 876 product descriptions observed in the daily expenditure diary, which provided a second textual source. Minor wording differences between the standardised expenditure dictionary and diary labels were reconciled only when product identity was unambiguous. Two additional fields in the standardised file, corresponding to unsalted nuts with positive expenditure and unambiguous naming, were directly classified as NOVA 1. No field with positive expenditure remained without an analytical status.

Fourteen categories had insufficient descriptions for a single NOVA assignment. Among them were powders for cocoa drinks, certain breakfast cereals, whipped cream, gelatin powder, vegetable shortening, light butter, heterogeneous dairy products, some mixed vegetable preparations and certain breads. These products were not forced into a category. They were coded as REVIEW in the primary analysis and were excluded from the denominator of classifiable spending. For each one, a plausible lower and upper NOVA group, defined from the official description, was established before the analysis. In this way, lower and higher sensitivity scenarios were generated for the national NOVA 4 share. The strategy follows the recommendations that propose to document the uncertainty of the classification process instead of hiding it within a categorical assignment (3, 22).

The sequence of construction, audit, and analysis is summarised in Figure 1. The figure is placed immediately after the description of the procedure because it represents complete traceability from official files to national estimates and statistical models.

**Figure 1:**
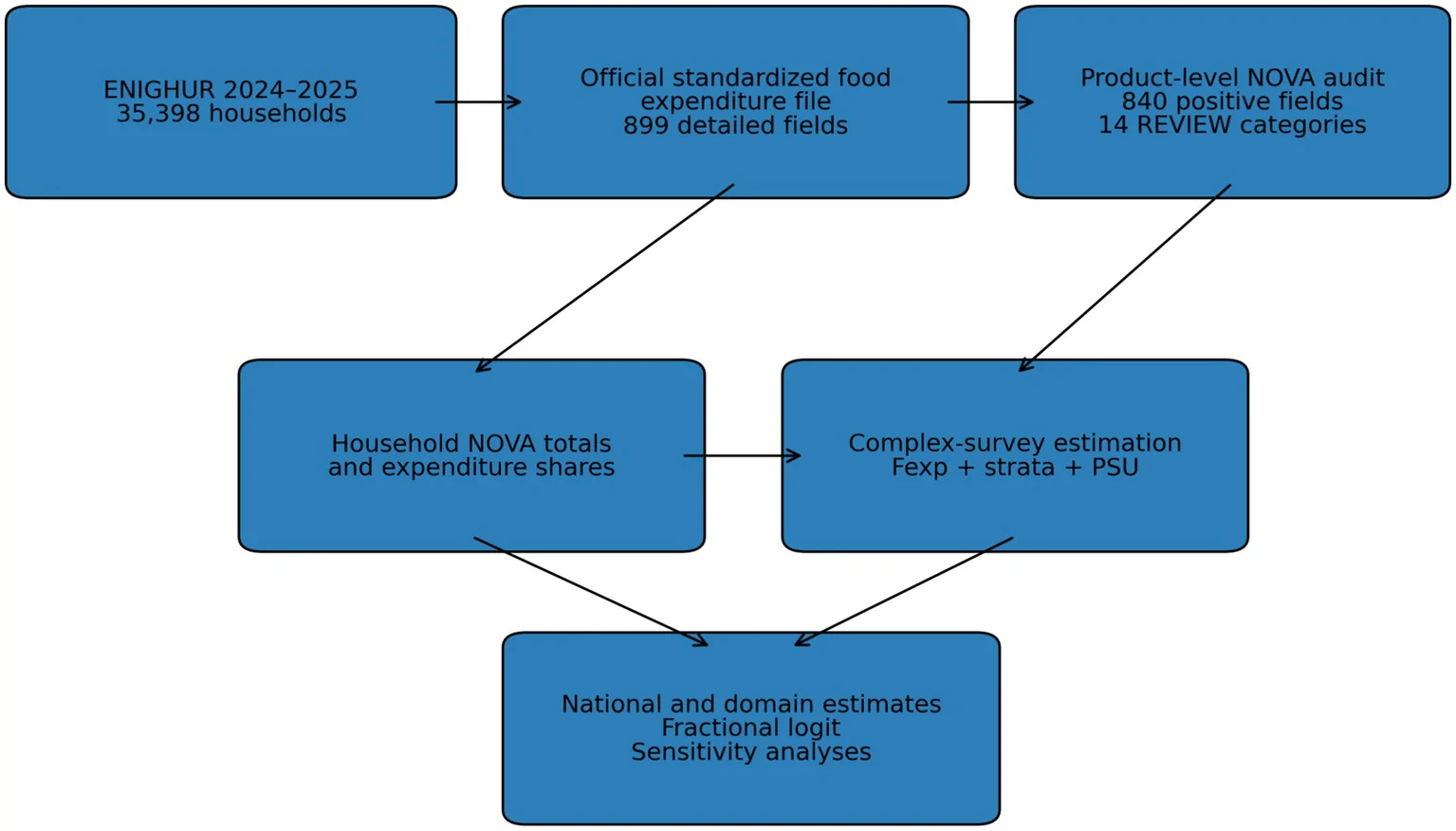
Reproducible flow of data processing and survey analysis used to derive household NOVA expenditure measures from the 2024–2025 ENIGHUR. The graphic content is kept in English to maintain the non-editable version of the figure.

### 2.4 Outcome variables and covariates

The main structure was defined in monetary terms. For household i, Gᵢg represented the standardised monetary expenditure assigned to the NOVA g group. The classifiable food expenditure Tᵢ corresponded to the sum of Gᵢ₁, Gᵢ₂, Gᵢ₃ and Gᵢ₄. NOVA 4 household share was defined as shown in Equation 1.


$$S_{i}₄=G_{i}₄/T_{i}$$ (1)

National shares were not calculated as the simple average of the percentages for each household. The national estimate for group g was defined as a ratio of weighted totals, so that households with different survey weights contributed according to their population representation as shown in Equation 2.


$$R(g)=\Sigma_{i}\;w_{i}G_{i}g/\Sigma_{i}\;w_{i}T_{i}$$ (2)

This estimate answers a food system question: what fraction of the classifiable food acquisition monetary expenditure of the population represented by ENIGHUR is allocated to each technological level of processing. As complementary results, an indicator of any NOVA 4 acquisition was constructed, equal to one when the household’s NOVA 4 expenditure was greater than zero; NOVA 4 per capita expenditure, obtained by dividing household expenditure by its number of members; product diversity within each NOVA group, defined as the number of detailed fields with a positive value; classification coverage, calculated as Tᵢ divided by Tᵢ plus REVIEW expense; and product contribution, calculated as the share of the weighted expenditure of each NOVA 4 field in the national NOVA 4 expenditure. None of these indicators were interpreted as energy contribution or nutrient intake.

The socioeconomic and territorial variables were selected before estimating the models. Current per capita income was the main continuous socioeconomic measure and was transformed using natural logarithm due to its skewness and the convenience of interpreting relative differences across a broad distribution. National income deciles were used in domain descriptive analyses. Household size was incorporated as a continuous covariate. Area of residence was coded as urban or rural. The natural region included Coast, Sierra, Amazon and Insular, with Coast as reference. The sex of the head was represented by an indicator of female headship. The age and educational level of the head were maintained in their official groupings, using the lowest code category as a reference. Periods 2 to 13 were represented by indicator variables and period 1 was used as a reference to absorb systematic variation during the annual survey cycle.

### 2.5 Estimation for complex surveys and variance

National and domain estimates simultaneously incorporated Fexp, stratum, and Identif_upm. For a ratio estimand R = Y/T, the linearized contribution of observation i was defined as uᵢ = wᵢ(yᵢ − Rtᵢ). Contributions were summed within each primary sampling unit. In each stratum h with mₕ observed primary units, the variance component was obtained from the quadratic deviations of the linearized totals of each unit with respect to its stratal mean and was multiplied by mₕ/(mₕ − 1). The total variance of the ratio corresponded to the sum of the stratal components divided by the square of the weighted denominator. Standard errors, 95% confidence intervals, and coefficients of variation were calculated with 3.292 design degrees of freedom, resulting from 3,425 primary units minus 133 strata. This procedure is consistent with the principles of inference for complex samples described by Lumley (25). The same domain estimators were used for income deciles, area, natural region, and survey period, avoiding recalculation of variances as if each domain constituted an independent sample.

### 2.6 Fractional model and adjusted predictions

The NOVA 4 share was modelled using fractional logit because the response was bounded between zero and one and included households with zero NOVA 4 expenditure. The model was specified as logit[E(Sᵢ₄|Xᵢ)] = β₀ + β₁ ln(incomeᵢ) + β₂ household sizeᵢ + β₃ urbanᵢ + β₄ female headshipᵢ + β₅ ln(incomeᵢ) × urbanᵢ + age categories + educational categories + natural region + period fixed effects. The income-by-area interaction was specified *a priori* because urban infrastructure and market density may modify the relationship between purchasing power and access to industrial formulations. Complete information was available for 34,570 households. Estimation incorporated survey weights. The robust covariance matrix was calculated from weighted score contributions aggregated by primary sampling unit and centred within strata, thereby preserving clustering and stratification in the sandwich estimator. The fractional specification follows Papke and Wooldridge (26), adapted to the survey design using the variance structure described above.

Adjusted shares were obtained using marginal standardisation. The 10th, 25th, 50th, 75th and 90th weighted percentiles of per capita income were identified. For each percentile, income and area were set at the values of interest, preserving the weighted empirical distribution of the rest of the covariates. Predicted means were averaged with Fexp. Standard errors were calculated with the delta method from the gradient of the standardised prediction with respect to the coefficients and the covariance matrix stratified by primary unit. This strategy makes it possible to interpret the income by area interaction directly on the NOVA 4 share scale, avoiding attributing isolated substantive meaning to the coefficient of the urban variable when there is an interaction.

### 2.7 Robustness and sensitivity analysis

As a robustness analysis, a weighted linear model was estimated for the continuous NOVA 4 share with the same specification of covariates and the same sandwich variance structure stratified by primary unit. The coefficients express absolute changes in the share and make it possible to verify that the direction of the associations does not depend exclusively on the logit link function. Joint Wald tests were performed for the age, education, natural region, and period blocks. Classification sensitivity was assessed by recalculating national NOVA 4 share after simultaneously assigning all items classified as REVIEW to their plausible lower category and, in a separate scenario, to their plausible upper category. All three scenarios used the same weighted totals ratio definition and the same survey variance estimator.

### 2.8 Complementary multivariate analysis of acquisition patterns

As a complementary analysis, weighted principal component analysis was applied to eleven official dimensions of household expenditure: cereals and cereal products; meats; fish and shellfish; milk, dairy products and eggs; oils and fats; fruits and nuts; vegetables, tubers, bananas and legumes; sugar, confectionery and desserts; prepared, instant and other foods; non-alcoholic beverages; and food services. Each aggregate was expressed per capita, transformed using ln(1 + x), and standardised using Fexp-weighted means and standard deviations. Components were obtained from the weighted covariance matrix and those with eigenvalues greater than one were retained and interpreted from their loadings. PCA was used only for descriptive dimension reduction and did not enter the inferential models for NOVA 4, which remained based on the survey-weighted fractional logit.

### 2.9 Reproducibility and quality control

Quality control was part of the base construction process. For each household, the sum of monetary expenditure assigned to NOVA 1, NOVA 2, NOVA 3, NOVA 4, REVIEW and EXCLUDED was compared with the official aggregate d1. The maximum absolute difference was 4.55 × 10^−13^ monetary units, a value compatible with floating point representation error and not with loss of information. Nationally, 99.676% of monetary expenditure within the classification scope was assigned to NOVA 1–4 in the primary analysis, while REVIEW accounted for 0.324%. Analytical variables, classification decisions, sensitivity limits, and statistical results were maintained in an auditable master database and in a separate statistical book. Calculations were run in Python 3.13.5 with NumPy 2.3.5, SciPy 1.17.0, and statsmodels 0.14.6. The sequence of official data, ranking map, master database and results can be reconstructed using the Identif_hog key and the detailed field identifiers.

### 2.10 Ethical considerations and data governance

The study used secondary, de-identified, publicly accessible microdata. No participants were recruited, there was no direct contact with the surveyed households, and no identifiable information was incorporated into the analytical files. The level of analysis was the household and the results are presented as population estimates, aggregate domains, or contributions of product categories. The official documentation and the original microdata remain in the custody of the National Institute of Statistics and Censuses of Ecuador.

## 3 Results

### 3.1 Analytical population and classification audit

The final analytical population comprised 35,398 households distributed in 3,425 primary sampling units and 133 strata. Of the 899 detailed food and non-alcoholic beverage fields in the standardised monetary file, 840 recorded positive expenditure in at least one household. All fields with a positive value received an analytical status of NOVA 1, NOVA 2, NOVA 3, NOVA 4, REVIEW, or EXCLUDED. Fourteen categories remained as REVIEW because the official name did not allow the degree of processing to be uniquely identified. The primary classification covered 99.676% of the standardised monetary expenditure within the analytical scope, while REVIEW accounted for 0.324%. Reconciliation by household with aggregate d1 was exact within numerical precision. Table 1 summarises the sampling structure and classification control used as the basis of all subsequent analyses.

**Table 1:** Analytical population and audit of product classification.

**Indicator**	**Worth**
Homes	35,398
Primary sampling units	3,425
Strata	133
Standardised food fields	899
Fields with positive expenditure	840
REVIEW categories	14
Expense classifiable within the scope	99.676%
REVIEW expenditure within scope	0.324%
Maximum reconciliation error vs. d1	4.55 × 10^−13^

REVIEW identifies categories whose official description did not allow for a single NOVA assignment. The reconciliation error corresponds to the maximum absolute difference between the sum of all technological states and the official aggregate d1.

### 3.2 National technological structure of acquisitions

The national expenditure structure was dominated by NOVA 1 foods. The weighted share was 72.91% with 95% CI of 72.55 to 73.26. NOVA 4 represented 12.25% with 95% CI of 11.91 to 12.58; NOVA 3 represented 11.74% with 95% CI of 11.55 to 11.93; and NOVA 2 reached 3.11% with 95% CI of 3.02 to 3.19. The coefficients of variation were 0.25% for NOVA 1, 1.40% for NOVA 2, 0.84% for NOVA 3 and 1.41% for NOVA 4, which shows adequate precision of the national estimates under the sampling design. The estimates are presented in Table 2 and their graphical representation is shown in Figure 2.

**Table 2:** Weighted national shares of monetary food expenditure by NOVA group.

NOVA group	Stake%	EE pp	Lower 95%CI	95% CI higher	CV%
NOVA 1	72.91	0.18	72.55	73.26	0.25
NOVA 2	3.11	0.04	3.02	3.19	1.40
NOVA 3	11.74	0.10	11.55	11.93	0.84
NOVA 4	12.25	0.17	11.91	12.58	1.41

Shares were estimated as ratios of weighted monetary totals. SE, standard error; pp, percentage points; CV, coefficient of variation.

**Figure 2:**
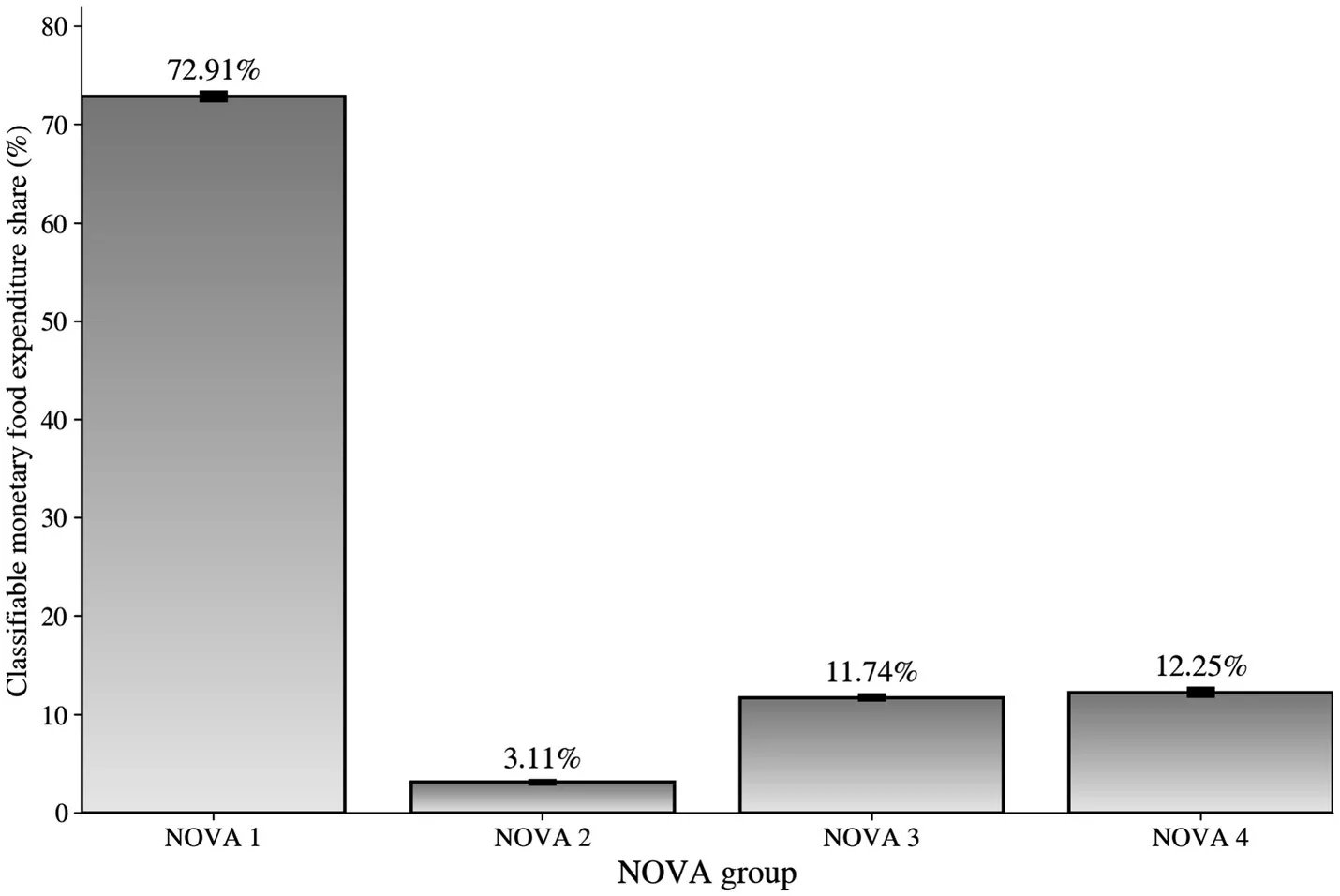
National composition of monetary food expenditure by NOVA group. Grey-gradient bars represent the estimated shares, and black vertical lines show design-based 95% confidence intervals.

Although NOVA 4 concentrated 12.25% of classifiable monetary expenditure, its presence was widespread in households. The weighted prevalence of any NOVA 4 expenditure was 79.99%. NOVA 4 standardised monetary expenditure per capita had a weighted mean of 5.64. The difference between share of expenditure and prevalence of acquisition indicates that ultra-processed products were present in a large proportion of household baskets, although their monetary value did not dominate the total acquisition. This pattern differs from a scenario in which a small subset of households concentrates most of the spending on industrial formulations.

### 3.3 Socioeconomic and territorial patterning

NOVA 4 share showed an upward gradient across national per capita income deciles. The values were 9.82% in decile 1; 10.78% in decile 2; 10.69% in decile 3; 11.10% in decile 4; 11.60% in decile 5; 12.31% in decile 6; 12.14% in decile 7; 13.68% in decile 8; 13.89% in decile 9; and 15.94% in decile 10. Minor reversals between adjacent deciles did not change the general trend. NOVA 4 expenditure per capita increased from 2.30 in the first decile to 10.71 in the tenth. Figure 3 presents the complete progression and its confidence intervals.

**Figure 3:**
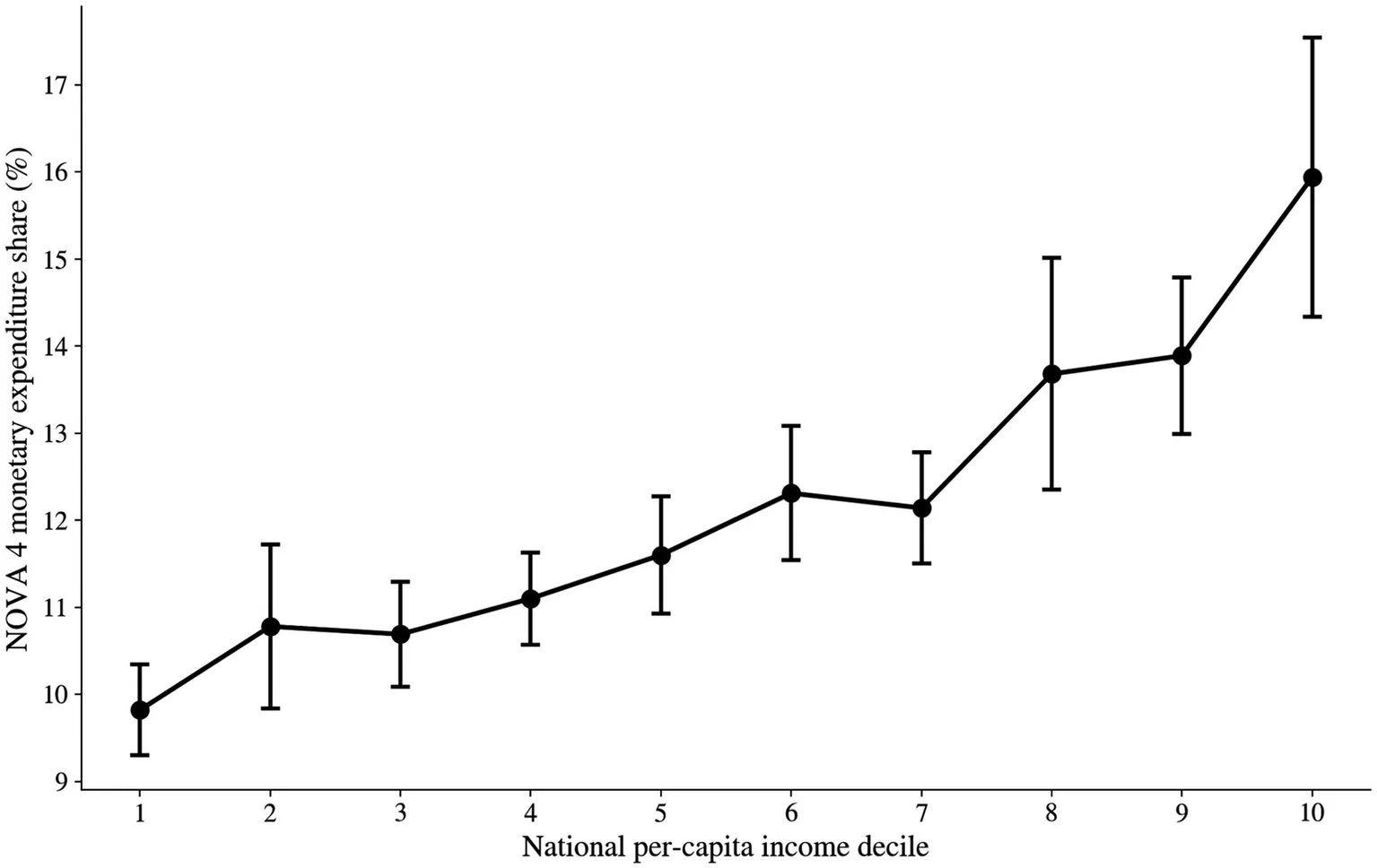
NOVA 4 weighted monetary share across national per capita income deciles. The line, markers, and 95% confidence intervals are presented in black; labels and axes are kept in English.

In the domain descriptive analysis, urban households allocated 12.75% of classifiable expenditure to NOVA 4, compared to 10.24% in rural households. The presence of any NOVA 4 acquisition was estimated at 81.74% of urban households and 74.63% of rural households. The corresponding per capita expenditure was 6.24 in the urban area and 3.81 in the rural area. NOVA 1 share was 72.64% in urban households and 73.98% in rural households. The urban–rural difference was expressed, therefore, both in the proportional structure of expenditure and in the probability of registering at least one NOVA 4 acquisition.

The differences between natural regions were smaller in amplitude than the income gradient, but were detectable. The NOVA 4 share was 12.20% in the Coast, 12.42% in the Sierra, 11.05% in the Amazon and 13.72% in the Island region. The proportion of households with any NOVA 4 expenditure varied from 75.22% in the Amazon to 85.98% in the Insular region. Per capita spending reached its highest value in the Insular region, with 14.16, and the lowest in Amazonia, with 3.91. NOVA 1 remained the main monetary component in all regions. Table 3 summarises the most relevant contrasts by income, area and region.

**Table 3:** Selected NOVA 4 acquisition indicators according to socioeconomic and territorial domain.

Domain	NOVA share 4%	Any NOVA 4% acquisition	NOVA 4 expenditure per capita
Income decile 1	9.82	77.45	2.30
Income decile 10	15.94	76.91	10.71
Urban	12.75	81.74	6.24
Rural	10.24	74.63	3.81
Coast	12.20	82.44	6.08
Saw	12.42	77.80	5.33
Amazon	11.05	75.22	3.91
Insular	13.72	85.98	14.16

The shares represent weighted spending ratios. Per capita spending corresponds to ENIGHUR’s standardised monetary measure.

### 3.4 Multivariate associations and adjusted NOVA 4 predictions

The fractional logit confirmed that the socioeconomic patterning of NOVA 4 could not be summarised by a single main effect of area of residence. The logarithm of per capita income presented a positive association with NOVA 4 share, *β* = 0.208, robust SE = 0.034, *p* < 0.001. Household size was also positive, *β* = 0.044, SE = 0.008, *p* < 0.001. The urban coefficient was negative on the model’s reference scale, *β* = −0.864, SE = 0.221, *p* < 0.001, while the income by urban interaction was positive, *β* = 0.162, SE = 0.039, *p* < 0.001. The presence of the interaction prevents interpreting the urban coefficient as an average contrast between areas. Female headship did not show an independent association after adjustment, *p* = 0.217. The selected coefficients are presented in Table 4. The full model is presented in Table S1.

**Table 4:** Selected coefficients from the weighted fractional logit model for household NOVA 4 monetary share.

Predictor	Coefficient	Robust SE	Lower 95% CI	Upper 95% CI	*p*
ln per capita income	0.208	0.034	0.141	0.275	<0.001
Household size	0.044	0.008	0.029	0.060	<0.001
Urban	−0.864	0.221	−1.297	−0.432	<0.001
Female-headed household	−0.032	0.026	−0.083	0.019	0.217
ln income × urban	0.162	0.039	0.084	0.239	<0.001
Sierra	−0.093	0.030	−0.153	−0.034	0.002
Amazonia	−0.080	0.036	−0.150	−0.010	0.025
Insular	−0.284	0.060	−0.401	−0.167	<0.001

Model additionally adjusted for age groups, educational level and survey periods. Costa and the group with the lowest code constitute the reference categories. The interpretation of the main effects of income and area must be done along with the interaction.

Wald tests showed heterogeneity between age groups, χ^2^ = 180.14 with 3 degrees of freedom, *p* < 0.001; natural regions, χ^2^ = 24.63 with 3 degrees of freedom, *p* < 0.001; and survey periods, χ^2^ = 107.65 with 12 degrees of freedom, *p* < 0.001. The educational block did not present an independent association after incorporating the rest of the variables, χ^2^ = 2.68 with 6 degrees of freedom, *p* = 0.848. The income by urban interaction remained significant in the joint model, χ^2^ = 16.75, *p* < 0.001. These results indicate that the acquisition pattern operates simultaneously across income, age, territory, and survey temporal variation, while the residual educational gradient is comparatively small when the other dimensions are controlled.

Standardised predictions clarified the interaction. At the 10th weighted percentile of per capita income, the adjusted NOVA 4 share was 9.81% for urban households and 10.66% for rural households. In P25 it was 11.12 and 11.43%, respectively. The curves crossed before the median. In P50 the predictions were 13.06% in urban and 12.52% in rural. In P75 they reached 15.44 and 13.77%, and in P90 they reached 18.05 and 15.07%. As seen in Figure 4, the adjusted contrast widened in the upper part of the income distribution, so the pattern does not correspond to a constant urban premium.

**Figure 4:**
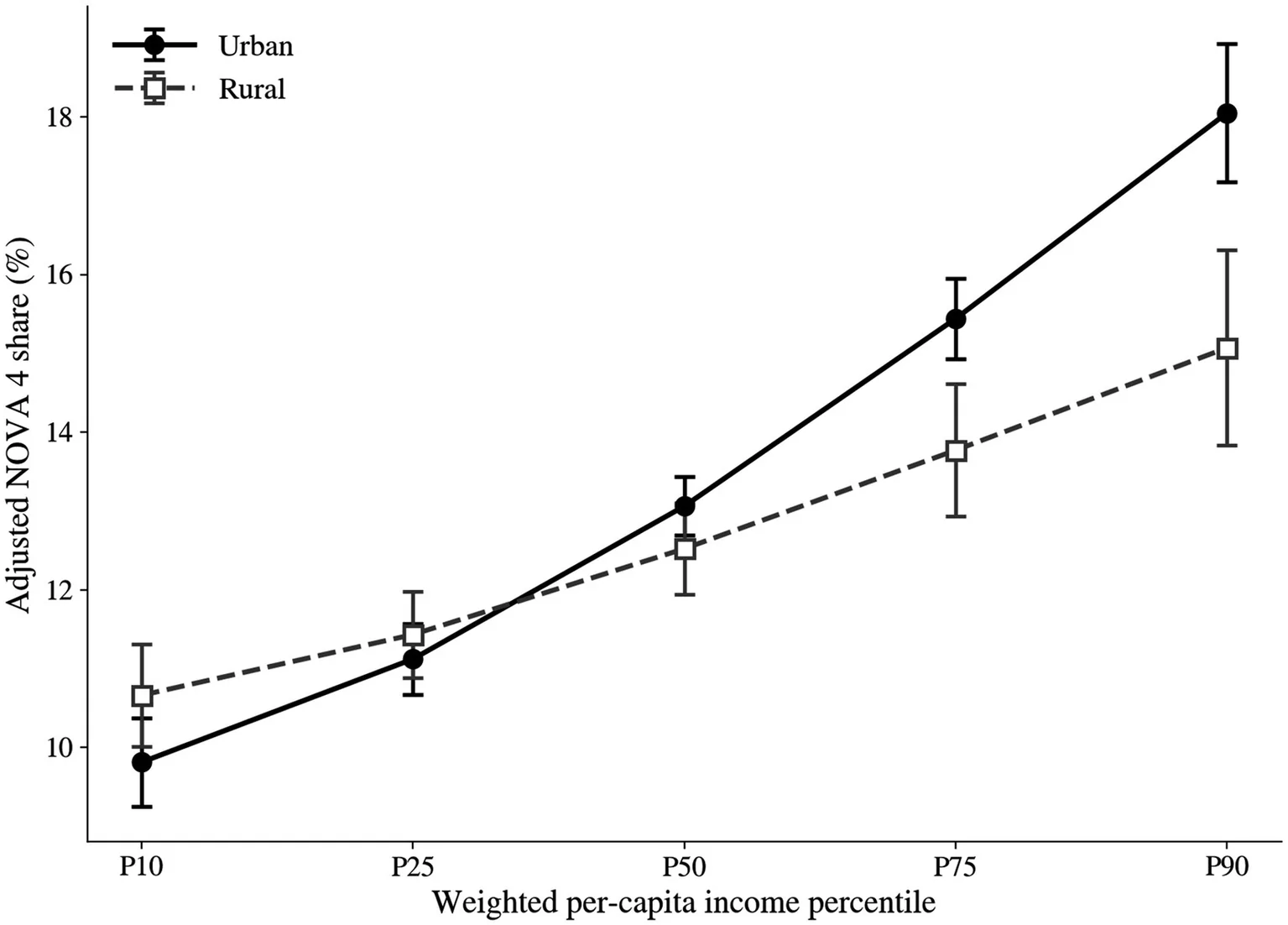
NOVA 4 shares adjusted by marginal standardisation across weighted percentiles of per capita income in urban and rural areas. Urban estimates are represented by a solid black line and filled circles; rural estimates by a dashed grey line and open squares. Vertical bars represent 95% confidence intervals.

### 3.5 Contributing products and classification sensitivity

The contribution analysis showed that NOVA 4 expenditure was distributed among numerous categories, although there was a relevant concentration in a subset of products. Carbonated soft drinks accounted for approximately 15.03% of weighted NOVA 4 monetary expenditure. Flavoured yoghurt contributed 8.17%, infant formulas 6.34%, commercial liquid or powder nutritional supplements 6.07%, sports and hydration drinks 4.66% and single-serving ice creams 4.34%. Crackers contributed 2.96%, artificial fruit drinks 2.50%, energy drinks 2.08% and white bread 1.99%. The four main categories concentrated around 35.6% of NOVA 4 expenditure and the top ten approximately 54.1%. The presence of infant formulas and nutritional supplements among the top contributors confirms that NOVA is a technological classification and not a product-by-product clinical hierarchy.

Classification sensitivity was small relative to the magnitude of the national estimate. When all fourteen REVIEW categories were simultaneously assigned to their plausible lowest category, the NOVA 4 share was 12.21% with 95% CI of 11.87 to 12.54. The primary estimate was 12.25% with 95% CI of 11.91 to 12.58. The upper scenario produced 12.53% with 95% CI of 12.19 to 12.87. The total interval between the two rating extremes was approximately 0.32 percentage points. The stability is explained by the low monetary weight of REVIEW and the concentration of uncertainty in a limited number of descriptions. The scenarios confirmed that the substantive interpretation of the national estimate did not depend on a single assignment of the ambiguous products.

### 3.6 Complementary multivariate structure of food expenditure

Weighted PCA retained three components explaining 54.44% of the weighted variance. PC1 explained 33.91% and showed positive loadings across most food groups purchased for the home, consistent with a general gradient of acquisition breadth. PC2 explained 11.06% and was dominated by food services (0.783) and non-alcoholic beverages (0.527). PC3 explained 9.47% and contrasted fruits and nuts, vegetables and meats with sugar, confectionery and desserts and with oils and fats. The detailed loadings are presented in Table 5. Because PCA was complementary, no inferential interpretation was assigned to component scores.

**Table 5:** Loadings of the first three weighted principal components of eleven dimensions of per capita food expenditure.

Dimension	PC1	PC2	PC3
Cereals and cereal products	0.344	0.003	−0.100
Meats	0.357	−0.112	0.269
Fish and shellfish	0.316	−0.131	−0.034
Milk, dairy products and eggs	0.352	0.122	0.176
Oils and fats	0.302	−0.175	−0.451
Fruits and nuts	0.318	0.043	0.450
Vegetables, tubers, bananas and legumes	0.392	−0.166	0.271
Sugar, confectionery and desserts	0.243	0.041	−0.581
Prepared, instant and other foods	0.266	−0.044	−0.170
Non-alcoholic drinks	0.230	0.527	−0.182
Food services	0.028	0.783	0.081
Eigenvalue	3.730	1.217	1.042
Explained variance (%)	33.91	11.06	9.47
Cumulative variance (%)	33.91	44.97	54.44

Per capita monetary variables were transformed using ln(1 + x), centred and scaled with weighting by Fexp. The sign of PC2 was oriented so that food services had a positive loading and that of PC3 so that fruits and nuts had a positive loading.

## 4 Discussion

### 4.1 Main findings and implications for the food system

The national estimates show that household monetary acquisition remained dominated by NOVA 1, while NOVA 4 was present in a large proportion of household baskets. These indicators answer different questions: share measures the fraction of classifiable food expenditure allocated to each processing group, whereas acquisition prevalence indicates whether any positive expenditure was recorded. Neither measure quantifies dietary intake. In particular, a higher NOVA 4 monetary share may reflect higher quantities, higher prices, a different mix of products, or a combination of these factors; it should not be interpreted as a proportional increase in biological exposure (9, 10).

The Ecuadorian monetary pattern is lower than energy-based estimates reported in some high-income settings, but those magnitudes are not directly comparable because the denominators differ (12). The result is best interpreted as a food-system baseline showing that minimally processed foods retain a central economic position while industrial formulations are widely integrated into household purchasing. The complementary PCA adds only a broader structural context: PC1 reflected general acquisition breadth, whereas PC2 was dominated by food services and non-alcoholic beverages.

### 4.2 Socioeconomic gradient and urban–rural interaction

The income gradient and its interaction with urban residence are the clearest socioeconomic findings. The adjusted predictions were similar at the lower income percentiles, crossed around the middle of the distribution and diverged thereafter, with a wider urban–rural difference at higher income. The model therefore does not support a constant urban effect; it shows that the association between income and NOVA 4 monetary share differs by area of residence.

An economic interpretation requires separating mechanisms that ENIGHUR can suggest from those it cannot identify directly. Greater purchasing power can relax budget constraints, while urban retail environments can provide broader assortments of packaged products, beverages, convenience foods and food services (27, 28). At the same time, monetary shares can change because of relative prices, product mix and household preferences, not only because of quantities purchased. The widening adjusted difference at higher income is therefore consistent with an interaction among purchasing power, retail access, prices and preferences, but the cross-sectional model does not separately estimate those channels.

Female headship was not independently associated with NOVA 4 monetary share after adjustment, but this result should be interpreted narrowly rather than as evidence that gendered household organisation is irrelevant to food expenditure. Öztornacı et al. (29), using household budget microdata from Türkiye, showed that gender interacts with income, education, employment stability, savings capacity and other household characteristics in shaping food-expenditure vulnerability. In the Ecuadorian model, the non-significant coefficient indicates only that female headship did not add an independent shift in NOVA 4 share conditional on the covariates included; gender-related pathways may operate through socioeconomic characteristics already represented in the model.

### 4.3 Territorial structure and product composition

Regional heterogeneity persisted after adjustment, but its causes were not tested. The Insular region showed the highest descriptive NOVA 4 share, acquisition prevalence and per capita expenditure, whereas Amazonia showed the lowest NOVA 4 share. Logistics, longer supply chains, tourism, retail structure and relative fresh-food costs are plausible contextual explanations for the Insular pattern, particularly given previous evidence of food-system change in Galapagos (17), but they should be treated as hypotheses for future research rather than findings of the present model.

The product-contribution profile offers a more direct technological interpretation. Carbonated soft drinks were the largest contributor to NOVA 4 expenditure, followed by flavoured yoghurt, infant formulas, nutritional supplements, sports drinks and ice cream. Because the top ten categories accounted for about 54% of NOVA 4 expenditure, surveillance can focus efficiently on markets where reformulation, packaging, serving size and pricing changes may materially alter the monetary structure. These contributions describe market composition and should not be read as a ranking of product-level health risk (3).

### 4.4 Methodological contribution and measurement validity

The methodological contribution is the separation of expenditure construction, technological classification and population inference. Official standardised expenditure was regrouped without changing the household total, 99.676% of in-scope expenditure received a definitive NOVA assignment, and the fourteen REVIEW categories altered the national NOVA 4 estimate only within a narrow sensitivity range. This auditability addresses known reproducibility problems in processing classifications (20, 21, 22). Equally important, the monetary estimand remains explicit: expenditure incorporates both quantity and price, so the findings concern how households allocate monetary resources within the acquisition basket, not energy intake or consumed food weight.

### 4.5 Implications for food policy and food technology in Ecuador

For food-system surveillance in Ecuador, the principal implication is to combine processing-based acquisition measures with existing nutritional and regulatory indicators rather than treat NOVA as a stand-alone policy metric. Ecuador’s traffic-light labelling and the NOVA 27 screener address nutritional information and individual exposure at different levels (14, 15, 18). ENIGHUR adds a population-level monetary baseline that can be repeated to assess whether the technological composition of household acquisitions changes across income groups, territories and policy periods. Product-level monitoring of beverages and other leading contributors could further connect expenditure surveillance with reformulation and price analyses.

### 4.6 Epidemiological context and value for surveillance

Epidemiological evidence provides the public-health rationale for monitoring NOVA 4, but it should not be used to convert the present expenditure estimates into measures of dietary exposure or disease risk. Controlled and prospective studies have linked higher ultra-processed food intake with energy intake and several health outcomes (30, 31, 32, 33). ENIGHUR measures a different construct. Its value is to identify the economic presence and distribution of industrial formulations in household acquisitions and to provide a baseline for longitudinal surveillance of how that structure changes.

### 4.7 Integration of findings and analytical outlook

Taken together, the evidence identifies three features of the Ecuadorian acquisition system: monetary predominance of minimally processed foods, a positive socioeconomic gradient in NOVA 4 share that becomes more urban at higher income, and modest but persistent territorial heterogeneity. Classification sensitivity was small, supporting the stability of the national estimate. The complementary PCA indicates that broader expenditure covariation contains a dimension dominated by food services and non-alcoholic beverages, but the principal inference remains the survey-weighted NOVA analysis. Future ENIGHUR rounds can use the same auditable classification framework to track whether these monetary patterns change over time.

## 5 Conclusion

The 2024–2025 ENIGHUR provides a reproducible national baseline for the monetary structure of Ecuadorian household food acquisitions by degree of processing. First, minimally processed foods remain the dominant component of classifiable expenditure, while NOVA 4 products are widely present in household baskets; these estimates describe monetary acquisition, not dietary intake. Second, NOVA 4 share rises across the income distribution and the adjusted urban–rural difference widens at higher income, indicating that purchasing power and territory jointly structure the monetary allocation to industrial formulations. Third, regional heterogeneity persists after adjustment and classification uncertainty has only a limited influence on the national estimate. These findings support the use of ENIGHUR for repeated food-system surveillance, particularly when processing-based indicators are interpreted alongside prices, product composition, nutritional information and existing public-health policy. The analysis therefore offers a transparent baseline for monitoring socioeconomic and territorial changes in Ecuador’s household acquisition environment.

## Data Availability

The original 2024–2025 ENIGHUR microdata and its official documentation are publicly accessible through the National Institute of Statistics and Censuses of Ecuador. The reproducible package of the study, which includes the processed data, the NOVA classification map, the analytical files, the derived statistical results, and the materials necessary to reproduce the analysis, is available on Zenodo: https://doi.org/10.5281/zenodo.21909198.
