# Supplementary material for: Industrial processing of Ecuadorian household food acquisitions according to NOVA, socioeconomic status and territory
Source: Front Public Health. 2026 Oct 12;14:1970719. doi: 10.3389/fpubh.2026.1970719 (PMC13696470; doi:10.3389/fpubh.2026.1970719)
Supplement: Supplementary file 1 [file Table_1.DOCX]

# **Supplementary analytical table**

Table S1 presents the full fractional logit model supporting the selected coefficients from Table 4. This table is cited here to maintain traceability between the main model and its parameters not shown in the body of the manuscript.

Table S1

Full weighted fractional logit model for NOVA 4 household monetary share.

| Predictor | Coefficient | Robust SE | Lower 95% CI | Upper 95% CI | p | exp(coefficient) |
| --- | --- | --- | --- | --- | --- | --- |
| Intercept | -2.4140 | 0.2175 | -2.8403 | -1.9877 | <0.0001 | 0.0895 |
| ln per capita income | 0.2079 | 0.0343 | 0.1407 | 0.2750 | <0.0001 | 1.2310 |
| Household size | 0.0444 | 0.0081 | 0.0285 | 0.0602 | <0.0001 | 1.0454 |
| Urban | -0.8645 | 0.2208 | -1.2972 | -0.4317 | <0.0001 | 0.4213 |
| Female-headed household | -0.0320 | 0.0259 | -0.0827 | 0.0188 | 0.2166 | 0.9685 |
| ln income × urban | 0.1616 | 0.0395 | 0.0842 | 0.2390 | <0.0001 | 1.1754 |
| Age group 2 | -0.2893 | 0.0770 | -0.4402 | -0.1384 | 0.0002 | 0.7488 |
| Age group 3 | -0.5741 | 0.0836 | -0.7379 | -0.4102 | <0.0001 | 0.5632 |
| Age group 4 | -0.8371 | 0.0879 | -1.0094 | -0.6647 | <0.0001 | 0.4330 |
| Education 2 | -0.0367 | 0.0637 | -0.1616 | 0.0882 | 0.5648 | 0.9640 |
| Education 3 | -0.0183 | 0.1033 | -0.2208 | 0.1842 | 0.8591 | 0.9818 |
| Education 4 | -0.0362 | 0.0660 | -0.1656 | 0.0932 | 0.5836 | 0.9645 |
| Education 5 | 0.0501 | 0.0871 | -0.1206 | 0.2208 | 0.5652 | 1.0514 |
| Education 6 | -0.0316 | 0.0916 | -0.2112 | 0.1480 | 0.7304 | 0.9689 |
| Education 7 | -0.0069 | 0.0742 | -0.1524 | 0.1385 | 0.9254 | 0.9931 |
| Sierra | -0.0933 | 0.0305 | -0.1530 | -0.0336 | 0.0022 | 0.9109 |
| Amazonia | -0.0804 | 0.0358 | -0.1505 | -0.0103 | 0.0246 | 0.9228 |
| Insular | -0.2840 | 0.0599 | -0.4014 | -0.1666 | <0.0001 | 0.7528 |
| Period 2 | -0.1137 | 0.0658 | -0.2427 | 0.0154 | 0.0842 | 0.8926 |
| Period 3 | -0.1925 | 0.0653 | -0.3206 | -0.0645 | 0.0032 | 0.8249 |
| Period 4 | -0.2851 | 0.0775 | -0.4370 | -0.1331 | 0.0002 | 0.7520 |
| Period 5 | -0.4746 | 0.0621 | -0.5963 | -0.3530 | <0.0001 | 0.6221 |
| Period 6 | -0.3933 | 0.0682 | -0.5271 | -0.2596 | <0.0001 | 0.6748 |
| Period 7 | -0.3246 | 0.0754 | -0.4723 | -0.1769 | <0.0001 | 0.7228 |
| Period 8 | -0.3250 | 0.0691 | -0.4605 | -0.1895 | <0.0001 | 0.7226 |
| Period 9 | -0.3700 | 0.0638 | -0.4951 | -0.2449 | <0.0001 | 0.6907 |
| Period 10 | -0.4377 | 0.0689 | -0.5727 | -0.3027 | <0.0001 | 0.6455 |
| Period 11 | -0.4205 | 0.0692 | -0.5560 | -0.2849 | <0.0001 | 0.6567 |
| Period 12 | -0.3983 | 0.0672 | -0.5300 | -0.2665 | <0.0001 | 0.6715 |
| Period 13 | -0.3823 | 0.0703 | -0.5201 | -0.2445 | <0.0001 | 0.6823 |

*Note.* The robust covariance matrix was constructed by aggregating scores by primary unit and centering within stratum. The reference categories correspond to code 1 for age, education and period, Coast for region and rural for area.
